# Promotion and suppression of autobiographical thinking differentially affect episodic memory consolidation

**DOI:** 10.1371/journal.pone.0201780

**Published:** 2018-08-03

**Authors:** Samarth Varma, Sander M. Daselaar, Roy P. C. Kessels, Atsuko Takashima

**Affiliations:** 1 Donders Institute for Brain, Cognition and Behavior, Radboud University, Nijmegen, The Netherlands; 2 Max Planck Institute for Psycholinguistics, Nijmegen, The Netherlands; Universite de Lyon, FRANCE

## Abstract

During a post-encoding delay period, the ongoing consolidation of recently acquired memories can suffer interference if the delay period involves encoding of new memories, or sensory stimulation tasks. Interestingly, two recent independent studies suggest that (i) autobiographical thinking also interferes markedly with ongoing consolidation of recently learned wordlist material, while (ii) a 2-Back task might not interfere with ongoing consolidation, possibly due to the suppression of autobiographical thinking. In this study, we directly compare these conditions against a quiet wakeful rest baseline to test whether the promotion (via familiar sound-cues) or suppression (via a 2-Back task) of autobiographical thinking during the post-encoding delay period can affect consolidation of studied wordlists in a negative or a positive way, respectively. Our results successfully replicate previous studies and show a significant interference effect (as compared to the rest condition) when learning is followed by familiar sound-cues that promote autobiographical thinking, whereas no interference effect is observed when learning is followed by the 2-Back task. Results from a post-experimental experience-sampling questionnaire further show significant differences in the degree of autobiographical thinking reported during the three post-encoding periods: highest in the presence of sound-cues and lowest during the 2-Back task. In conclusion, our results suggest that varying levels of autobiographical thought during the post-encoding period may modulate episodic memory consolidation.

## Introduction

In the recent testing-effect/retrieval practice literature, the retrieval of recently learned items from memory has been shown to strengthen memory traces (see reviews by [1, 2]). However, even in the absence of conscious rehearsal, interference-free post-encoding environments like sleep or quiet wakeful rest can lead to significant improvements in the retention of recently acquired memories through a process of memory consolidation [3–5]. During such offline periods, the hippocampus triggers repeated reactivation of neural activity patterns, which code the prior learning experience [6, 7], gradually strengthening the associated memory trace [8, 9].

At the same time, there are several factors that can distort or interrupt the consolidation of recently acquired memories [10, 11]. Firstly, it is evident that contextually overlapping memory processing, such as cue-overload or AB-AC paradigm [12], can cause forgetting due to similarity in the content of initial and subsequent learning (i.e., A-B association is weakened after learning A-C association). Secondly, interference can also arise from cognitive processes that deal with information that is unrelated or dissimilar to prior learning. For example, the retention of a wordlist is significantly reduced when learning is followed by engagement in tasks, such as the spot-the-difference task, mental arithmetic, tone-detection, picture search or the viewing of video clips, as compared to a short period of quiet wakeful rest [13–15]. Finally, forgetting may also occur due to internally generated thoughts [14]. Note that, in the absence of tasks that require intentional control of thoughts or directed attention towards stimulus processing, a resting mind generally tends to wander to images, voices and feeling, etc. [16, 17]. However, in the presence of external stimulation, this tendency can be exaggerated, with consequences of interference to ongoing memory consolidation [14]. For example, when participants are presented with a wordlist followed by a 9-minute rest period, interspersed with ten short familiar sound-cues (e.g., a cat’s meow, which could trigger participants to think about things related to cats), memory retention of the studied wordlist significantly drops as compared to when the study phase is followed by 9 minutes of quiet wakeful rest [14]. Behavioral reports further indicate that such environmental cues could trigger retrieval of cue-associated memories from one’s personal past and/or imagination of a future scenario, even in the absence of explicit instructions to do so [14, 18].

The forgetting of studied materials observed in these tasks can be associated with the interruption of activities that aid the consolidation process. Such interruption may arise from novel memory encoding or retrieval, associated with sensory stimulation from the environment or from autobiographical thinking [13–15, 18]. Mednick and colleagues [11] and Wixted [10] proposed that this retroactive interference is caused when novel encoding usurps limited hippocampal resources that are otherwise engaged in consolidating previously encoded memories. They suggest that there is a tradeoff between resources allocated towards ongoing memory consolidation and novel goal-directed memory processing (keeping track of the current environment, attending to salient stimuli and maintaining a logical stream of thought). Under this assumption, consolidation of recently acquired memories could suffer interference when the post-encoding period is filled with novel episodic memory processing -such as autobiographical thinking—which is unrelated to the encoded material [14]. Limiting such autobiographical thoughts during the post-encoding period could free up episodic memory resources for ongoing consolidation, thereby reducing interference effects.

Preliminary evidence in support of this idea comes from our recent research where we demonstrated that post-encoding engagement in a modified 2-Back task leads to the same degree of memory retention as in a quiet wakeful rest [19]. Specifically, our results showed that the degree of memory retention does not differ when the post-encoding period is filled with 9 minutes of quiet wakeful rest or 9 minutes of a 2-Back task. This result was replicated across six experiments, involving different memoranda (word-picture pairs, wordlists and faces), task designs (3-Back and a difficulty-adjusted 2-Back task) and memory tests (free-recall and recognition). On the one hand, the degree of offline consolidation during an n-Back-task may have been lower as compared to a quiet wakeful rest period, as participants may have had less opportunity to engage in learning-related memory processes (e.g., automatic reactivation and spontaneous or intentional rehearsal of studied words, pictures or faces) during the n-Back tasks. On the other hand, the continuous attentional demands of the n-Back task might have reduced the type of mindwandering/autobiographical thinking that can occur during wakeful rest and has been shown to interfere with consolidation [14]. Moreover, the n-Back task could also have reduced experimental/environmental stimulation or autobiographical thinking associated with the use of familiar sounds, stories or pictures used in common interference tasks [5, 13, 14].

Numerous functional magnetic resonance (fMRI) studies have shown reduced processing in the hippocampus during a 2-Back task, as compared to a fixation baseline [20] or a non-memory guided sensorimotor baseline task, such as a 0-Back task [21–23], suggesting lower involvement of this structure in performing the 2-Back task. This relative change in hippocampal activity could arise from a reduction in episodic memory processes normally associated with mental imagery and autobiographical thinking [20, 24]. Patients suffering from episodic memory disorders due to, for example, schizophrenia or temporal lobe epilepsy, are also able to execute the 2-Back task, but their reduced performance seems to arise from a failure to successfully deactivate medial temporal lobe (MTL) structures, including the hippocampus [25–27]. But this does not seem to be the case for all working-memory (WM) tasks. Some fMRI studies have also found evidence for the recruitment of MTL structures in WM tasks, like, Delayed Match-to-Sample (DMS) and Sternberg paradigms for encoding relational items, sequences and maintenance of multiple items [28–30]. Relative to the 2-Back, these tasks place low demands on the continuous updating of WM and high demands on temporary storage and maintenance of presented items for delayed recognition, which could lead to sustained neural activation in the MTL [27]. Additionally, as compared to DMS, Sternberg and interfering tasks, such as mental arithmetic, the 2- and 3-Back tasks reported in Varma et. al [19], had a short ISIs (~ 800ms) and employed trial-by-trial feedback to induce constant deployment of attention and self-monitoring. Therefore, the 2-Back task demands might act as a cognitive barrier against interference from thoughts that are unrelated to the previously encoded material. In contrast with a post-encoding period that triggers autobiographical thinking or other episodic memory processes, engaging in a 2-Back task during the post-encoding period may spare limited episodic memory resources for consolidation processes. However, this has never been tested behaviorally within-subjects and within the same experimental design.

These independent, cross-study observations motivated our hypothesis in the current study that tasks promoting autobiographical thinking during the post-encoding period are detrimental to consolidation, as compared to tasks that suppress autobiographical thinking. Similar to previous studies [14, 19], our testing paradigm consisted of three blocks of incidental encoding of wordlists, each followed by a 9-minute delay (consolidation) period. This period either involved a quiet wakeful rest (baseline), a rest period interspersed with familiar sounds (‘rest+sounds’ condition, promoting autobiographical thinking), or a 2-Back task (suppressing autobiographical thinking), in a counterbalanced order. Following the three encoding-delay periods, there was a delayed free-recall test of all studied wordlists. We compared the effect of these three periods on the memory retention of words learned prior to these delays. An experience-sampling questionnaire was also added at the end of the experiment to test whether the degree and nature of post-encoding thoughts was related to the degree of memory consolidation in each delay condition. Following our hypothesis, and as shown previously by Craig et al., (2014) [14], we predicted that the rest+sounds condition would show greater forgetting, as compared to the rest condition due to interference from autobiographical thoughts cued by the familiar sounds. Furthermore, we hypothesized that the 2-Back task may not cause interference to consolidation compared to the rest+sounds condition, since the task demands allow little room for autobiographical thinking. A finding in favor of our hypothesis would motivate a reexamination of the role of spontaneous autobiographical thinking in memory consolidation and the brain states necessary for consolidation or interference to occur.

## Material and methods

We combined the paradigms used in the two studies described above [14, 19] to investigate whether a post-encoding period filled with a 2-Back task is better than an autobiographical-thinking task for consolidation of the studied wordlist. The procedure consisted of three blocks of incidental encoding of wordlists, each followed by a 9-minute delay period [14, 19], involving quiet wakeful rest, rest with sounds, or a 2-Back task, in a counterbalanced order, and ending with a delayed free-recall test of the wordlists. We compared the effect of these three periods on the retention of words learned prior to these delays. At the end of the experiment, participants also completed an experience-sampling questionnaire.

### Participants

Assuming η_p_^2^ = 0.19 from our prior work [19] we calculated that 34 participants were necessary for reliable power (1-β = 0.95) [31]. However, counterbalancing for within-subject factors employed in previous research [14] required the number of participants to be 36 (across six rotations of order). Of the recruited 36 participants, six were removed from the study due to inattentiveness to the task (i.e., not complying with the instructions) or indiscriminate or inaccurate button presses, resulting in poor performance on the 2-Back task (d-prime > 2-*SD* below the group average). Six more participants were recruited to replace the outliers, while ensuring counterbalancing of order. In total, 42 native Dutch-speaking, healthy students (40 women, *M*_age_ = 21.69, *SD* = 2.57, see limitations section) were recruited from the Radboud University student pool, of which 36 (34 women, *M*_age_ = 21.75, *SD* = 2.56) were considered for analysis after outlier removal. After receiving written and oral instructions from the experimenter, all participants gave written informed consent in accordance with the Declaration of Helsinki. At the end of the experiment, participants received course credits or monetary compensation. This study was reviewed and approved by the Ethics Committee of the Faculty of Social Sciences of Radboud University.

### Encoding lists

Forty-five commonly used Dutch nouns were recorded in the voice of a native speaker of the Dutch language. These words were chosen to have minimal semantic relatedness but were matched on frequency and concreteness. From these 45 words, 3 lists of 15 words each were prepared and assigned an equal number of times to the three conditions, across the six counterbalanced orders.

### Procedure

**Fig 1:**
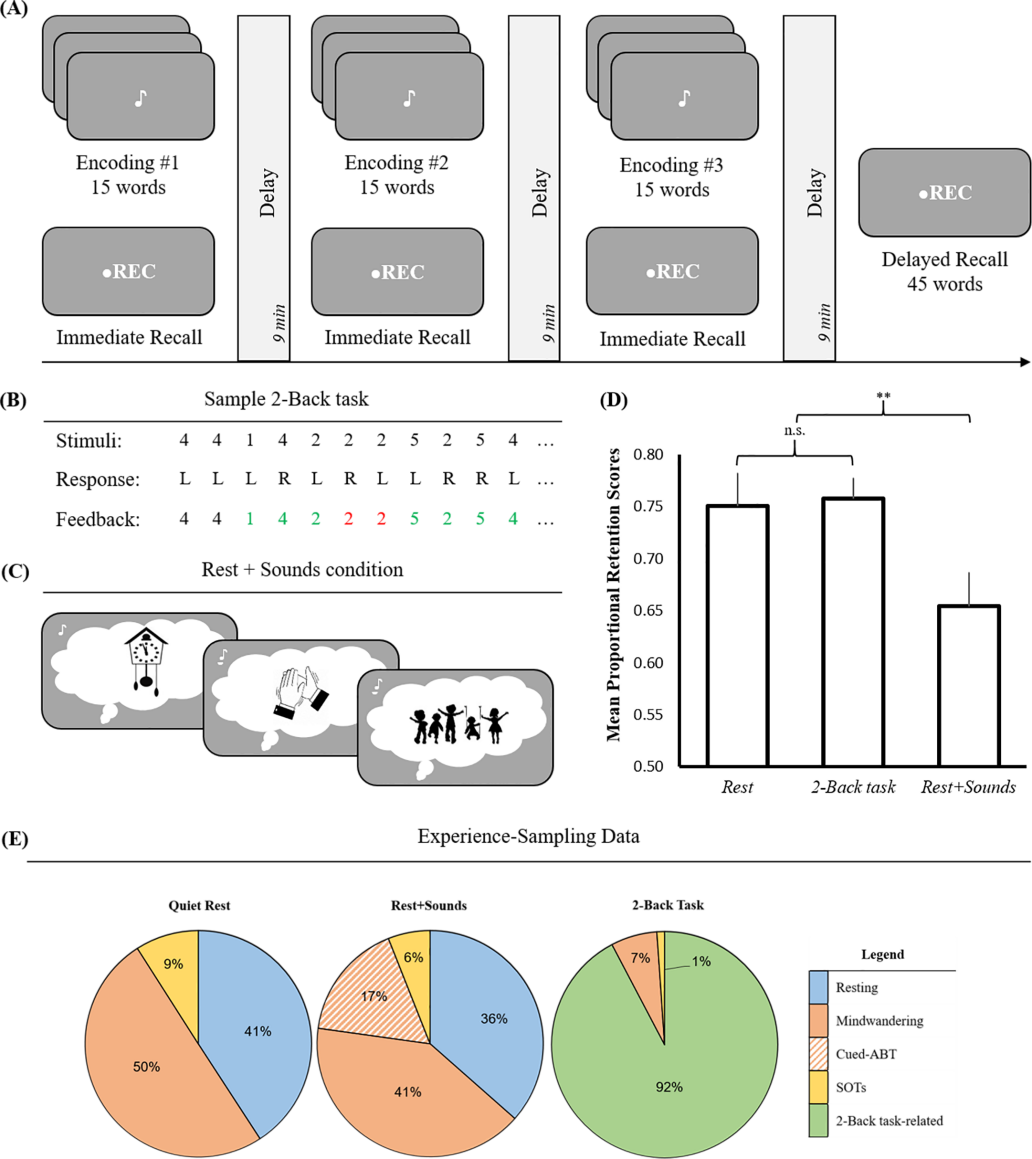
Experimental design and results. (A) General experimental design involved memorizing and recalling a list of 15 words in three incidental encoding blocks. Each encoding block was followed by a delay period occupied either by quiet wakeful rest (rest condition) or rest interspersed with familiar sounds (rest+sounds condition), or a 2-Back task (2-Back condition) in a counterbalanced order across subjects. The duration of these delay periods was set to 9 minutes. At the end of the three encoding-delay sessions, an unexpected delayed recall test measured memory retention of all 45 words. (B) Sample sequence in the 2-back task. ‘L’ and ‘R’ correspond to ‘Left’ and ‘Right’ arrow key, one for target and the other for non-target response, indicating sample responses to the 2-Back stimuli. Highlighted numbers indicate correct (green) or incorrect responses (red). (C) Rest+sounds condition depicting sample autobiographical past/future thoughts triggered by sound cues. (D) Results show a significant reduction in retention when learning was followed by the rest+sounds condition, as compared to the 2-Back condition. No significant difference was observed between the 2-Back and rest conditions. Error bars represent +1 *SEM*. (E) Results from the post-experimental experience-sampling questionnaire, showing the average proportions of thoughts related to various mental activities during the three delay periods (here ‘SOTs’ stands for Stimulus-Oriented Thoughts and ‘ABT’ stands for Autobiographical Thoughts).

The experiment was divided into three blocks, one for each condition (Fig 1A). Every block consisted of an encoding session, followed by an immediate recall test and a 9-minute delay (consolidation) period. Across the three blocks, the 9-minute delay periods were occupied either by a quiet wakeful rest (rest condition), a rest period interspersed with familiar sounds (rest+sounds condition), or a 2-Back task (2-Back condition), in a counterbalanced order across participants. There were no breaks between successive blocks. At the end of the third block, we measured memory retention of the three lists using an unexpected free-recall test (delayed recall). During both immediate and delayed recall tests, participants could recall as many words as possible, in any order. A mobile device was used to record responses during these tests, which were scored offline. The experiment was designed using the PsychoPy presentation software [32]. Stimuli and PsychoPy files for the encoding, rest+sounds and 2-Back conditions can be found under S1 Dataset and S1 Protocol, respectively.

#### Encoding task

During the encoding block, a list of 15 words was presented aurally, one word every 2 s. Participants were instructed to memorize the given wordlist, with the expectation of a quick test of retention immediately following the presentation. An ‘immediate recall’ test was then conducted to obtain a score for initial memory retention, before the 9-minute delay period began.

#### 2-Back task

One of the three encoding sessions was followed by a delay of 9 minutes, during which participants engaged in a 2-Back task involving numbers. The design of the 2-Back task was identical to that in our previous experiments (see Exp. 1–2, 5–6 in [19]. For each trial, a random number (between 1–5) was displayed in the middle of the screen for a maximum of 3 s. Participants were instructed to press the “right” key if this number was the same as the one displayed two trials earlier, or press the “left” key otherwise. Upon responding, the number turned green or red for 300 ms, indicating whether the judgment was correct or incorrect, respectively (Fig 1B). Such trial-by-trial feedback was aimed at motivating participants to be more attentive towards the task in order to achieve optimal performance. No other item or information was displayed on the screen to avoid distraction. At the beginning of the experiment, participants were acquainted with the demands of the task via a short practice session.

#### Quiet rest

Similar to previous research [13, 14], during the rest condition, subjects remained in the room for 9 minutes, during which a fixation-cross remained on the screen. After dimming the lights, the experimenter left the room to ‘prepare the next part of the study’. During this time, participants were instructed to rest quietly while remaining seated.

#### Rest with sound-cues

The design of the rest+sounds condition was adapted from Craig et al. [14]. For this condition, participants were presented with 10 audible stimuli (4–5 s long) during the post-encoding delay period of 9 minutes. These stimuli consisted of sounds encountered in everyday life (e.g. ‘clapping’, ‘playground’, ‘clock’, etc.) that may trigger memories from one’s personal past or the imagination of a future scenario (Fig 1C), but did not overlap with words in the study lists. The sound cues were randomly spaced apart (20 s– 70 s), and the task lasted 9 minutes in total. Participants were instructed to rest quietly while sounds would be played to keep them awake. No instructions were given to identify the sounds nor engage in any autobiographical thinking during this period. Lights in the room remained dim during this condition.

### Experience-sampling questionnaire

At the end of the experiment, participants completed a computer-based non-descriptive experience-sampling questionnaire. The purpose of the questionnaire was to a) assess proportions of thoughts during the delay period that were related to the encoding task (rehearsal of the words) or unrelated (spontaneous mindwandering or autobiographical thinking related to the cued sounds) and, b) verify that subjects experienced overall more autobiographical thinking during the rest+sounds condition relative to the rest condition. Subjects were asked to answer a question regarding the rest delay period, which was, “What % of your thoughts were related to each of the following activities: a) Resting/Meditation/Relaxation/Absence of any specific thoughts, b) Thoughts about past/present/future events, c) Words you learnt prior to the delay period, and d) Other thoughts (please provide examples).” Hereafter, the proportion of thoughts related to past/present/future events is referred to as ‘mindwandering’. The proportion of spontaneous thoughts related to or intentional rehearsal of previously learnt words is referred to as stimulus-oriented thoughts or SOTs. Mental activities related to meditation, relaxation or absence of any specific thoughts were classified under proportion of thoughts related to ‘rest’ to distinguish them from mindwandering. For the 2-Back delay period, the question regarding ‘% of Thoughts related to Resting/Meditation…’ was replaced by ‘% of Thoughts related to the number task’. For the rest+sounds delay period, the question regarding ‘% of Thoughts about past/present/future events’ was split into two: participants were asked about thoughts related to the sound-cues (autobiographical thoughts), versus thoughts unrelated to the sound-cues (mindwandering). In summary, for each delay period, participants had to indicate the proportion of thoughts that were related to various mental activities such as rest, SOTs, mindwandering, 2-Back task or autobiographical thinking related to sound-cues, adding up to a total of 100%. In case of a non-zero response to the proportion of “Other thoughts” during a delay period, its value was added to the appropriate thought-category of the associated delay period, depending on the examples provided by the participant in the questionnaire.

### Analyses

We calculated a proportional retention score for each wordlist, by dividing the number of words recalled during the delayed recall test by those originally recalled during the immediate recall test. Where the delayed recall score exceeded the immediate recall score (which was true for 1 participant in the 2-Back condition), the proportional retention score was capped at 1. In order to confirm that baseline memory performance did not differ across the three wordlists, we ran a repeated measures (RM)-ANOVA with immediate recall scores as within-subject dependent variables. A second RM-ANOVA was conducted on the proportional retention scores to test for the effect of the three delay periods on memory performance. ‘Order’ was added as a between-subjects variable to test whether the main effects persisted in the presence of any residual interaction between the order of the encoding-delay blocks and memory performance, despite counterbalancing. From the experience-sampling questionnaire data, rest and rest+sounds conditions were compared on the proportion of thoughts related to SOTs, rest and mindwandering, using paired-samples t-tests. Since the critical difference between the rest and rest+sounds conditions was the presence of the sound cues, we tested whether there was a difference in memory retention between these conditions that correlated with the difference in overall degree of autobiographical thinking experienced during each condition (i.e., % of mindwandering in the case of rest condition vs. % of mindwandering + cued autobiographical thoughts in the case of rest+sounds condition). Finally, we also tested for a correlation between thought proportions reported in the questionnaire and associated memory performance in each condition by computing Spearman’s rho, *r*_*s*_. All results were analyzed using IBM SPSS 23, and alpha was set at 0.05 throughout.

## Results

Immediate recall scores (rest: *M* = 10.67, *SD* = 2.39; 2-Back: *M* = 10.97, *SD* = 2.20; rest+sounds: *M* = 10.78, *SD* = 2.42) did not differ significantly between the three encoding blocks, *F*(2, 70) = 0.033, *p* = 0.96, η_p_^2^ = 0.001, indicating that the quality of memory encoding matched across the three wordlists.

The results from the second RM-ANOVA showed a significant main effect of the delay periods, in terms of the proportional retention scores (rest: *M* = 0.75, *SD* = 0.19; 2-Back: *M* = 0.76, *SD* = 0.11; rest+sounds: *M* = 0.66, *SD* = 0.19), *F*(2, 60) = 5.32, *p* = 0.007, η_p_^2^ = 0.15. In line with previous work [14], we found that the rest condition was significantly better than the rest+sounds condition, *t*(35) = 2.28, *p =* 0.029, CI = [0.01, 0.18], while the rest and 2-Back conditions did not differ [19], *t*(35) = -0.22, *p* = 0.83, CI = [-0.08, 0.06]. As predicted, planned t-tests showed that memory performance was higher when learning was followed by the 2-Back condition, than by the rest+sounds condition, *t*(35) = 2.82, *p =* 0.008, 95% confidence intervals (CI) = [0.03, 0.18] (see Fig 1D). We also found a trend towards statistical significance in the interaction between delay condition type and delay condition order, on the proportional retention scores associated with the delay conditions (*F*(10, 60) = 1.94, *p* = 0.06, η_p_^2^ = 0.25). A post hoc LSD test showed that this interaction effect is related to a difference between the conditions in two out of six order groups (*p* < 0.06), both of which ended with the 2-Back condition. No differences were found using other post hoc tests. Results of a paired t-test within the first order group (rest/rest+sounds/2-Back condition) reflected the main findings of the study: rest and 2-Back conditions did not differ from one another (*p* = 0.85), but both had a higher retention than the rest+sounds condition (*p* = 0.068 and *p* = 0.078, respectively). However, in the second order group (rest+sounds/rest/2-Back condition), only the 2-Back condition showed higher retention than the rest+sounds condition (*p* = 0.04). Independent t-tests on individual conditions showed no differences across order groups. These results suggest a slight advantage to memory retention in delay conditions that occur either at the beginning or at the end, compared to the delay condition that occurs in the middle. It is also possible that the rest+sounds condition in the second group (rest+sounds/rest/2-Back condition) casts proactive interference on the rest condition occurring next. However, no reliable conclusions can be drawn from this analysis due to a small sample size (*n* = 6) within each order group.

Average performance on the 2-Back task across subjects reached a mean accuracy of 92% (*SD* = 3%), with a mean d-prime of 2.58 (*SD* = 0.51) and a reaction time of 0.79 s (*SD* = 0.19 s), revealing that the participants adhered to the guidelines of the 2-Back task. Supporting information on individual and collated recall performance can be found under S2 Dataset.

The experience-sampling questionnaire data (see Fig 1E) showed that participants reported mindwandering during both rest and rest+sounds conditions, while a majority (22 out of 36) also reported autobiographical thoughts related to sound cues during the rest+sounds condition. Regarding stimulus-oriented thoughts or SOTs, 25 participants reported to have intentionally or spontaneously thought about the learnt words during the rest condition. This included 21 participants in the rest+sounds condition and 5 in the 2-Back condition. Four participants reported SOTs across all delay periods. Upon excluding these four participants, the main effect of the delay periods remained significant.

Planned t-tests showed that the proportion of SOTs (rest: *M =* 9.02%, *SD* = 10.47, rest+sounds: *M =* 5.97%, *SD* = 7.54) was significantly higher during the rest condition than the rest+sounds condition (*t*(35) = 2.38, *p* = 0.02). The degree of SOTs during the rest condition deviated from normality with a positive skew and kurtosis, with a Shapiro-Wilk test showing very high significance (*p* < 0.001). There was also a significant correlation between SOTs with memory retention score obtained from the rest condition (Spearman’s Rho, *r*_*s*_ = 0.41, *n* = 36, *p* = 0.012). This suggests that SOTs contributed to memory retention in the rest condition (see S1 Fig). Furthermore, upon removing two outliers in the SOT score, the correlation remained significant (*r*_*s*_ = 0.48, *n* = 34, *p* = 0.004). The degree of SOTs during the rest+sounds condition also deviated from normality with a positive skew and kurtosis, Shapiro-Wilk test (*p* < 0.00). However, unlike the rest condition, no correlation was observed between the degree of SOTs and memory retention associated with the rest+sounds condition (*r*_*s*_ = 0.03, *n* = 36, *p* = 0.86) (see S2 Fig). Moreover, the difference in degree of SOTs between the rest and rest+sounds conditions did not correlate significantly with the difference in the retention scores between the rest and rest+sounds conditions (*r*_*s*_ = - 0.13, *n* = 36, *p* = 0.47). This finding suggests that the degree of SOTs alone cannot explain the benefit of rest over rest+sounds condition.

Secondly, the proportion of mindwandering (i.e., spontaneous thoughts related to past/present/future) (rest: *M =* 50.13%, *SD* = 20.33, rest+sounds: *M =* 40.69%, *SD* = 19.09) was also significantly higher during the rest condition than during the rest+sounds condition (*t*(35) = 2.78, *p* = 0.009). But the proportion of mindwandering during rest was significantly lower when compared with the proportion of overall autobiographical thinking (mindwandering + cued-autobiographical thinking) during the rest+sounds condition (*M =* 57.5%, *SD* = 18.65; *t*(35) = 2.058, *p* = 0.047). This result suggests that in the presence of familiar sound cues, participants did in fact engage in a higher degree of autobiographical thinking during the rest+sounds condition than during the rest condition. However, the difference between the degree of mindwandering during the rest condition and the overall autobiographical thinking during the rest+sounds condition, did not correlate significantly with the difference in the retention scores between the rest and rest+sounds conditions (*r*_*s*_ = 0.132, *n* = 36, *p* = 0.44).

Third, the proportion of rest-related thoughts did not differ between the two rest conditions (*t*(35) = 1.20, *p* = 0.238).

Finally, during the 2-Back condition, ‘Task-related’ thoughts occupied the majority of this delay period (*M =* 92.3%, *SD* = 9.72), leaving little room for rest, SOTs or mindwandering as compared to other delay periods. No other questionnaire measures were found to correlate with memory performance in any of the conditions.

## Discussion

Independent studies have shown that a period of quiet wakeful rest and a 2-Back task demonstrate comparable levels of memory consolidation, as measured later by behavioral memory performance [19], whereas a rest period involving cues for autobiographical thinking interferes when compared to a period of quiet wakeful rest [14]. Accordingly, the degree of autobiographical thinking in the post-encoding period might differentially affect memory consolidation. In this study, we tested this hypothesis in a within-subject design involving a 2-Back task that suppresses autobiographical thinking and a rest+sounds condition that promotes autobiographical thinking using sporadic cues of familiar sounds. These conditions were compared against a period of quiet wakeful rest serving as a baseline. We successfully replicated the results of previous studies [14, 19] and provided supporting evidence for the idea that a post-encoding period involving a 2-Back task is better for the fate of memory consolidation than a rest period that triggers autobiographical thinking. Results from experience-sampling questionnaire substantiate our findings by showing that interference to episodic memory consolidation is related to the promotion and suppression of autobiographical thinking during the post-encoding period. We discuss these findings in separate sections below.

### Rest vs. rest+sounds condition

Neurobiological studies have shown that a period of quiet wakeful rest is beneficial for consolidation by allowing higher opportunity for the automatic reactivation of recently acquired memories and minimal interference from external stimulation [6–11]. However, recent findings also have shown that when the rest period contains intermittent cues promoting autobiographical thinking, the ongoing consolidation of the studied material suffers interference [14]. In the current study, we replicated this finding by demonstrating a significant difference in the memory retention of words learnt prior to a period of quiet wakeful rest (rest condition) and a period of rest interspersed with familiar sound cues (rest+sounds condition). Data from the experience-sampling questionnaire (Fig 1E) supports our findings by showing that the rest+sounds condition, which had the lowest memory performance, was also associated with the highest proportion of overall autobiographical thinking (mindwandering + cued-autobiographical thinking). The lack of difference in the proportion of rest-related thoughts (such as meditation, relaxation, or absence of any specific thoughts), ruled out any disparity in the degree of rest experienced in either condition. However, the difference in memory performance between the rest and rest+sounds conditions did not correlate with any differences in the proportions of thought categories reported in the questionnaire. It is possible that other factors also contributed to the reduction in memory performance associated with the rest+sounds condition, or that the questionnaire did not tap into factors responsible for interference to consolidation (see limitations section). There is ample evidence from neuroimaging studies suggesting that in order to reconstruct/relive past events and to create stimulations of novel future scenarios, autobiographical thinking draws on the same elaborate episodic memory processing as necessary for consolidation of recently acquired information [33–35]. In the same vein, our results show that the presence of familiar sound cues led to a marked increase in spontaneous autobiographical thinking as compared to a period of quiet wakeful rest. As a result, it is likely that the limited episodic memory resources need to be reallocated from ongoing consolidation to novel memory processing demands of concurrent goals like autobiographical thinking [7, 8].

### Rest vs. 2-Back condition

Unlike the effect of interference observed with the use of an autobiographical thinking task (rest+sounds condition) [14], the 2-Back condition did not differ from the rest condition in terms of subsequent memory performance. Our own research [19] has shown that post-encoding engagement in a 2-Back task leads to the same degree of memory consolidation as quiet wakeful rest, irrespective of memoranda, task designs and memory measures. While a rest period is considered ideal for consolidation of episodic memories, possibly due to higher chances of rehearsal [2] and a higher likelihood for automatic reactivation of the studied items [11], the issue of the similarity in memory performance across a 2-Back task and a quiet wakeful rest delay period requires further exploration of these different brain states.

Firstly, it is unclear why, unlike previous studies [13, 14], a large number of our participants experienced stimulus-oriented thoughts or SOTs during the delay periods. Regardless, data from the experience-sampling questionnaire indicates that quiet wakeful rest may be superior to the 2-Back task in terms of the opportunity it provides for SOTs. Conversely, in the case of the 2-Back condition, participants reported minimal SOTs as compared to other delay conditions, wherein the majority of thoughts were occupied by ‘Task-related’ (2-Back related) activities (Fig 1E). Furthermore, our analysis indicates that the degree of SOTs reported during the rest condition correlates with the memory retention of items learnt prior to this period. However, the difference in the permissibility of SOTs may not be crucial in discriminating these post-encoding periods. Previous studies have also reported beneficial effects of post-encoding rest period even when the majority of participants did not engage in SOTs such as rehearsal [13, 14], or when the encoding material could not be rehearsed [5, 8]. Moreover, prior work has also shown that the benefit of rest periods on memory consolidation is unrelated to the extent of SOTs during rest [36, 37]. These findings indicate that it is not SOTs but, rather, memory reactivation that drives the benefit of memory consolidation during rest [38]. Nonetheless, our results indicate that in the presence of verbalizable material, SOTs could benefit memory retention (similar to testing-effects, see [1]), especially when the post-encoding delay period involves quiet wakeful rest.

Nonetheless, rest is also associated with numerous complex processes, which involve both spontaneous and intentional processing of thoughts that are cued by both internal and external events [39, 40]. As stated previously, these resting-state processes may not only involve rehearsal of the studied items that promote consolidation, but also autobiographical thoughts that could interfere with the consolidation of studied items by reallocating resources necessary for such consolidation [11]. Similarly, it could be argued that the high proportion of thoughts related to mindwandering during the rest period (*M* = 50%) may have elicited novel encoding/retrieval that could have interfered with memory consolidation of the wordlist. Experience-sampling questionnaire data and 2-Back task performance showed that mindwandering during the 2-Back condition (e.g., experiences during task-engagement such as stress/boredom, time-monitoring) was minimal (*M* = 6%), since participants were continuously engaged in the task (RT = 0.7 s, mean accuracy = 92%).

In conclusion, although a 2-Back task may be disadvantageous due to reduced chances of automatic reactivation and SOTs, its non-episodic nature and continuous cognitive demands could benefit consolidation by reducing chances of interference from mindwandering or autobiographical thinking.

### Rest+sounds vs. 2-Back condition

The critical difference between the 2-Back and rest+sounds conditions lies in the nature of memory processing required by these tasks. The stimuli and performance of a 2-Back task is working-memory dependent [22, 23], and, possibly, allows episodic memory resources to be utilized for consolidation of the studied wordlist, as opposed to autobiographical thinking [33–35], such as in the rest+sounds condition. Results from the experience-sampling questionnaire clearly show that the mere presence of familiar cues in the environment led to a sizeable increase in the overall amount of autobiographical thinking, experienced by the participants during an otherwise restful state (Fig 1E), whereas the continuous attentional demands during the 2-Back task suppressed spontaneous task-unrelated mindwandering.

Regarding the possibility of stimulus-oriented thoughts or SOTs, the 2-Back task allowed for little to no opportunity (*M* = 1%), whereas around 6% of thoughts during the rest+sounds condition were associated with SOTs. Nonetheless, we suspect that any advantage that SOTs might have had on wordlist consolidation during the rest+sounds condition over the 2-Back condition, may have been undermined by the high amount of autobiographical thinking (*M* = 57%) that transpired in the rest+sounds condition (see Fig 1E).

On the basis of our results, we cannot conclude whether the benefit of the 2-Back condition over the rest+sounds condition was a direct consequence of the suppression of autobiographical thoughts during the 2-Back task. However, by replicating previous findings [14, 19] within a single experiment, we do provide evidence that reduced levels of autobiographical thinking is associated with better memory consolidation. Future neuroimaging work is necessary to further examine the effect of a 2-Back task on areas involved in autobiographical thinking and their interaction with consolidation of episodic memories.

### Limitations

Even though we have been discussing the possible role of the hippocampus as promoting/interfering with the ongoing memory consolidation of recently acquired memories, our study design was purely behavioral in nature. Thus, neuroimaging studies are warranted to corroborate any neural processing that we assume to be taking place.

Given the absence of explicit instructions to engage in autobiographical thought, participants may have attempted to find associations between the sound stimuli and the words in the list, or remember them for a future test. Moreover, we did not assess whether our participants were familiar with the sound cues in the rest+sounds condition. Although very unlikely, given the nature of the sounds used (everyday sounds such as ‘clapping, ‘playground’, ‘clock’ etc.), it is still possible that one or more of the sounds may have surprised some participants and/or caused unconscious encoding of the unrecognizable sounds. Accordingly, we cannot completely rule out the possibility that the interference observed during the rest+sounds condition may be affected by factors other than autobiographical thinking.

Although we deferred the experience-sampling questionnaire to the end of the experiment to avoid any uncontrolled interference effects (similar to psychometric tests, see [41]), it is likely that this delay might have reduced accuracy or reliability in reports of thought proportions during the delay periods. If the questionnaire was administered immediately after the delay, we might have been able to evaluate whether the content or richness of autobiographical thoughts affected memory performance [14].

A final limitation of this study is that the gender distribution of the recruited sample included more women than men. We do not foresee any gender-related differences in terms of general episodic memory consolidation, but we acknowledge that the findings can only be generalized to a female population.

## Conclusion

Neuroimaging research has shown that during downtimes like quiet wakeful rest, there is a boost in memory consolidation, probably due to a high degree of consolidation promoting processes, such as automatic reactivation of the memory traces [10, 11]. However, our present results indicate that rest may involve some aspect of interference from memory processing, such as autobiographical thinking that may hamper the ongoing consolidation process. Replicating previous research [14], we demonstrate that this effect is exacerbated in the presence of explicit environmental cues that could trigger novel encoding/retrieval of memories and future imaginations. In contrast, engaging in a demanding 2-Back task during the post-encoding period can reduce interference to consolidation of studied material by suppressing spontaneous autobiographical thinking, similar to our previous study [19]. Being able to compare these effects in a within-subject design, we conclude that the degree of autobiographical thinking modulates memory consolidation. In line with previous work [14], we find that tasks that promote autobiographical thinking by means of environmental cues can lead to interference to memory consolidation, whereas tasks that suppress autobiographical thinking by engaging in continuous working memory processing benefit memory consolidation [19]. It can therefore be concluded that our ability to maintain goal-directed episodic memory processing can result in a partial interruption of ongoing consolidation of recently acquired memories [10]. This tradeoff might be a necessary feature of memory-processing mechanisms that manifests itself in the allocation of our limited episodic memory processing resources. From an educational point of view, future research could study whether engaging in n-Back-like games or skill-development tasks, after a study session, could be used as learning aids that minimize forgetting of recently learnt classroom materials in the presence of environmental stimulations.

## Supporting information

S1 Dataset Stimulus dataset. Zip file containing three excel spreadsheets: 1) encoding wordlists, 2) numbers presented during the 2-Back task and, 3) labels for sound cues presented during the rest+sounds condition.
(ZIP)

S2 Dataset Recall dataset. Excel file containing 1) immediate and delayed recall data of individual participants, 2) Immediate Recall scores across participants and 3) Proportional Retention scores across participants. Information on age, gender, counterbalancing, and statistical tests is also added.
(XLSX)

S1 Fig Correlation between SOTs and memory retention during the rest condition. X-axis corresponds to the proportion of stimulus-oriented thoughts (SOTs) related to the encoded wordlist during the rest condition. Y-axis corresponds to the proportional memory retention of the wordlist encoded prior to the rest condition. Plot represents the correlation between these measures (Spearman’s Rho, *r*_*s*_ = 0.41, *n* = 36, *p* = 0.012) where each dot represents a single participant and the dotted line represents best-fit linear trendline.
(TIF)

S2 Fig Correlation between SOTs and memory retention during the rest+sounds condition. X-axis corresponds to the proportion of stimulus-oriented thoughts (SOTs) related to the encoded wordlist during the rest+sounds condition. Y-axis corresponds to the proportional memory retention of the wordlist encoded prior to the rest+sounds condition. Plot represents the correlation between these measures (Spearman’s Rho, *r*_*s*_ = 0.03, *n* = 36, *p* = 0.86), where each dot represents a single participant and the dotted line represents best-fit linear trendline.
(TIF)

S1 Protocol PsychoPy experiment files. Zip file containing three PsychoPy files corresponding to the encoding task, 2-Back task and the rest+sounds condition.
(ZIP)
