# Supplementary figures and images for: Promotion and suppression of autobiographical thinking differentially affect episodic memory consolidation
Source: PLoS One. 2018 Aug 3;13(8):e0201780. doi: 10.1371/journal.pone.0201780 (PMC6075762; doi:10.1371/journal.pone.0201780)

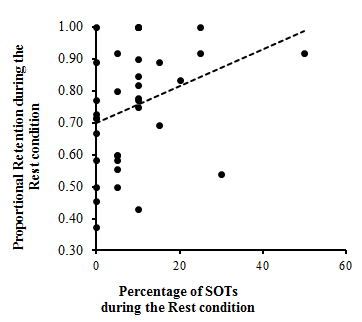

Supplement: S1 Fig — X-axis corresponds to the proportion of stimulus-oriented thoughts (SOTs) related to the encoded wordlist during the rest condition. Y-axis corresponds to the proportional memory retention of the wordlist encoded prior to the rest condition. Plot represents the correlation between these measures (Spearman’s Rho, rs = 0.41, n = 36, p = 0.012) where each dot represents a single participant and the dotted line represents best-fit linear trendline. (TIF) [file pone.0201780.s003.tif]

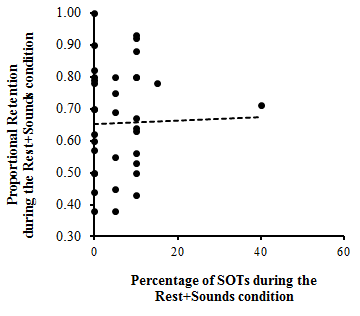

Supplement: S2 Fig — X-axis corresponds to the proportion of stimulus-oriented thoughts (SOTs) related to the encoded wordlist during the rest+sounds condition. Y-axis corresponds to the proportional memory retention of the wordlist encoded prior to the rest+sounds condition. Plot represents the correlation between these measures (Spearman’s Rho, rs = 0.03, n = 36, p = 0.86), where each dot represents a single participant and the dotted line represents best-fit linear trendline. (TIF) [file pone.0201780.s004.tif]
